# Supplementary figures and images for: Sourdough starters exhibit similar succession patterns but develop flour-specific climax communities
Source: PeerJ. 2023 Oct 4;11:e16163. doi: 10.7717/peerj.16163 (PMC10559884; doi:10.7717/peerj.16163)

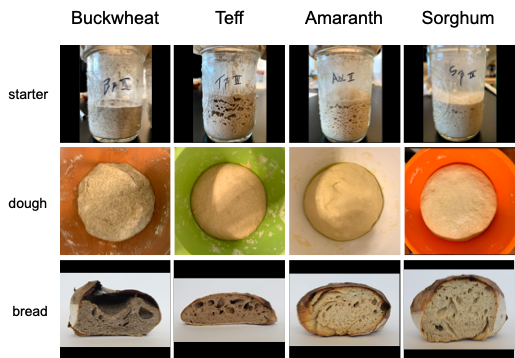

Supplement: Supplemental Information 4 — After 14 days we used a standard recipe to make bread from each starter, to verify leavening and indulge in an informal bread tasting to celebrate the completion of data collection. [file peerj-11-16163-s004.png]

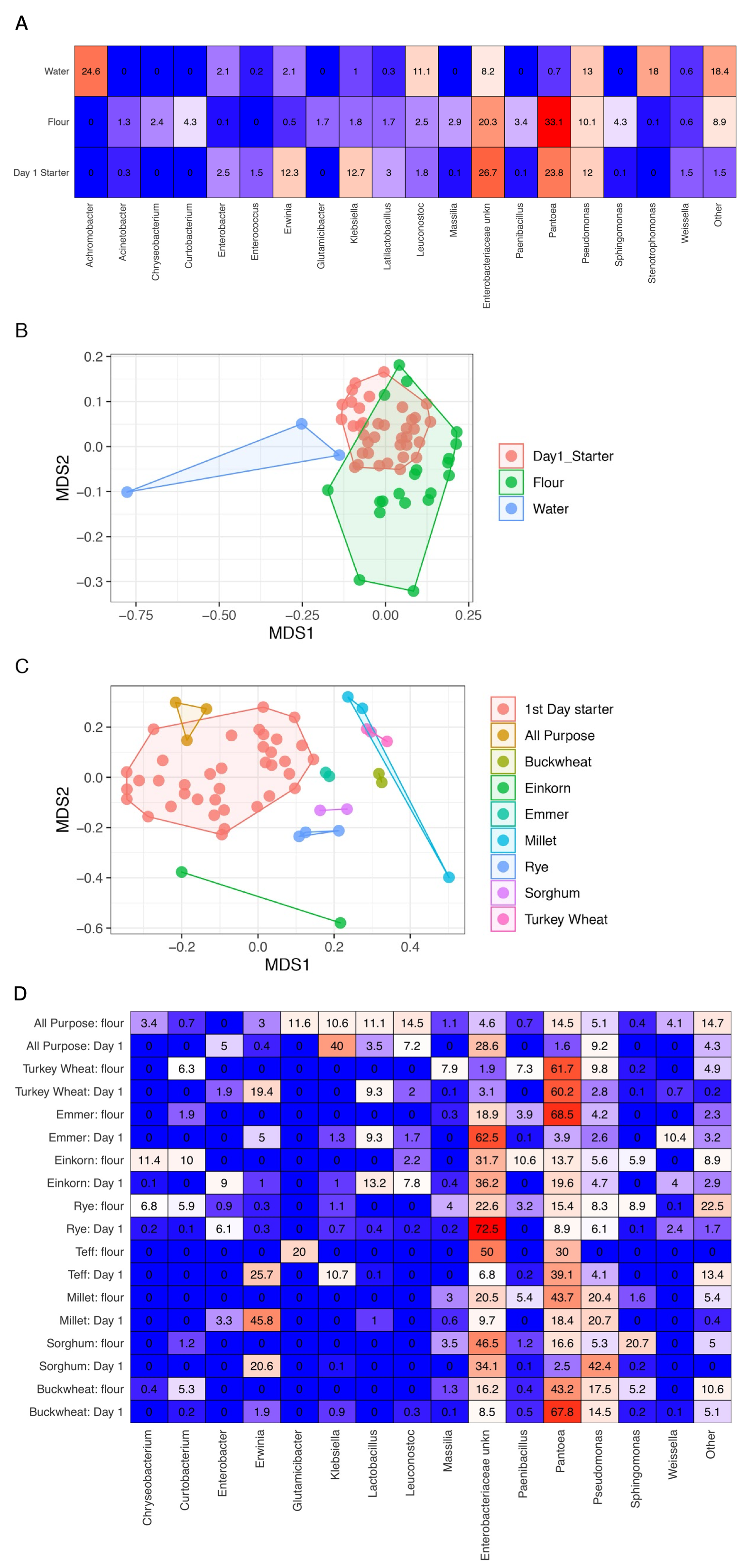

Supplement: Supplemental Information 5 — (A) Bar chart comparing average relative abundance (%) of bacterial taxa detected in water, flour, and day 1 starters. (B) Bray-Curtis NMDS plot comparing all water, flour, and day 1 starter samples. Water samples cluster separately. Overlap between flour and Day 1 samples is minimal and driven by a few flour samples. (C) Bray-Curtis NMDS plot comparing all flour inputs versus day 1 starter samples. (D) Heat map comparing the average relative abundance (%) of bacterial taxa detected in each type of flour, compared to the day 1 starters grown from that flour type. [file peerj-11-16163-s005.png]

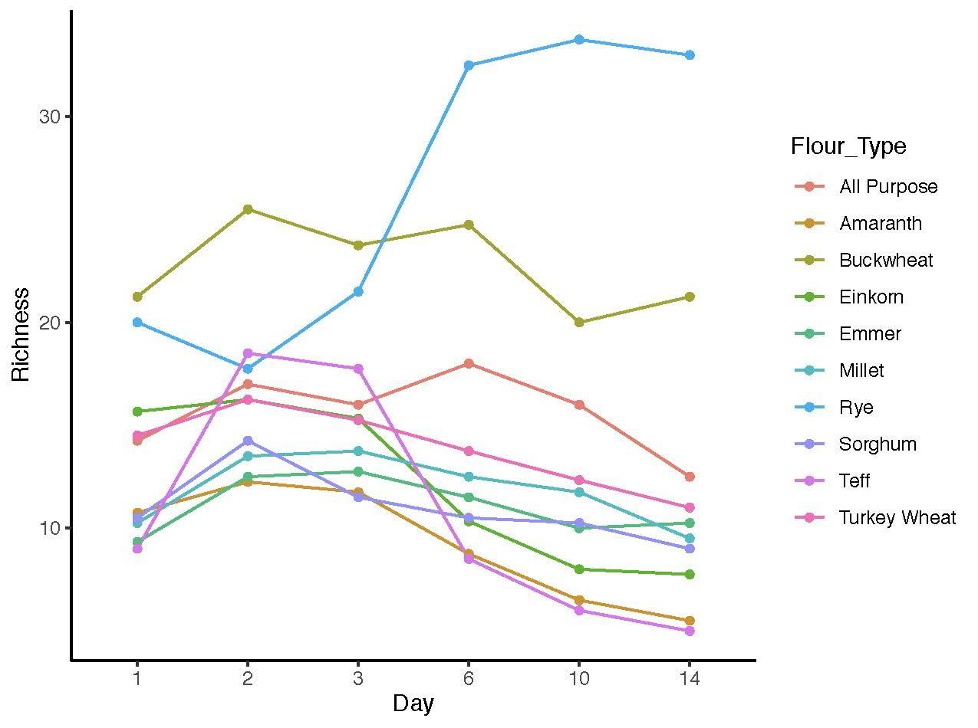

Supplement: Supplemental Information 6 — Values are averaged by flour type on days 1, 2, 3, 6, 10, and 14. [file peerj-11-16163-s006.png]

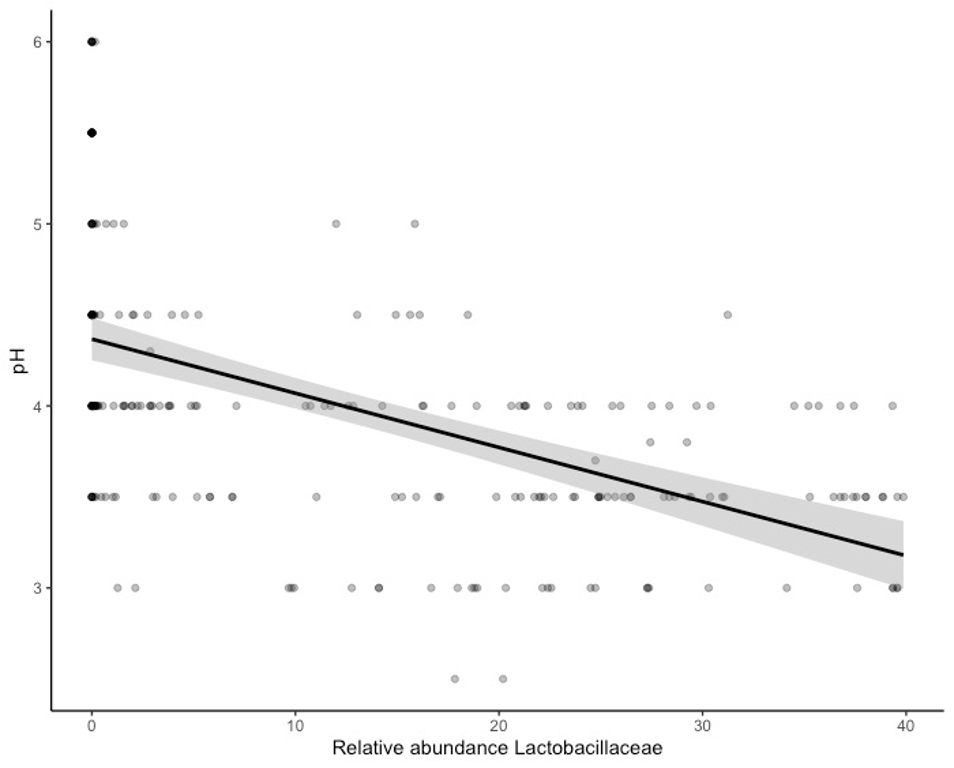

Supplement: Supplemental Information 7 — Starters were sampled at 1, 2, 3, 6, 10, and 14 days elapsed and grown from all purpose, amaranth, buckwheat, einkorn, emmer, millet, rye, sorghum, teff, and turkey wheat flour. Pearson correlation = −0.5321391. [file peerj-11-16163-s007.png]

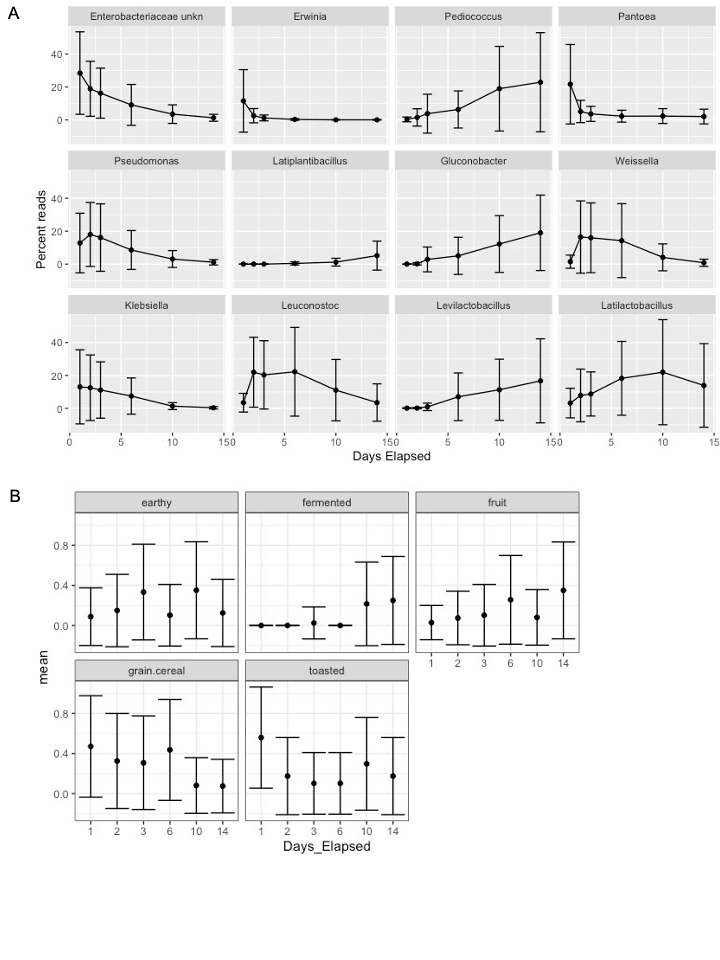

Supplement: Supplemental Information 8 — (A) Bacterial genera and (B) aromas that were identified as significantly different at different time points, across flour types. Significance determined based on Kruskal-Wallis tests after Bonferroni correction, p < 0.05. [file peerj-11-16163-s008.png]

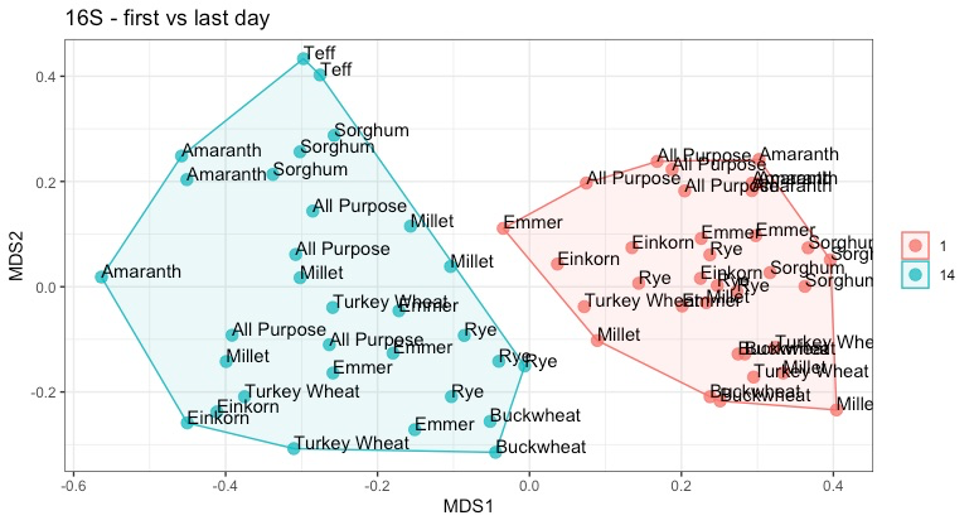

Supplement: Supplemental Information 9 — Each sample is labeled by flour type (all purpose, amaranth, buckwheat, einkorn, emmer, millet, rye, sorghum, teff, turkey wheat). [file peerj-11-16163-s009.png]

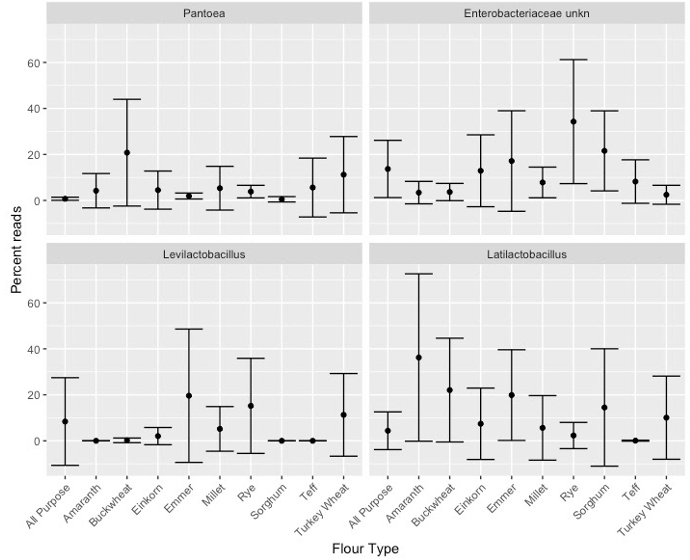

Supplement: Supplemental Information 10 — Significance determined based on Kruskal-Wallis tests after Bonferroni correction, p < 0.05. [file peerj-11-16163-s010.png]
